# Epigenetic Characterization of the *FMR1* Gene and Aberrant Neurodevelopment in Human Induced Pluripotent Stem Cell Models of Fragile X Syndrome

**DOI:** 10.1371/journal.pone.0026203

**Published:** 2011-10-12

**Authors:** Steven D. Sheridan, Kraig M. Theriault, Surya A. Reis, Fen Zhou, Jon M. Madison, Laurence Daheron, Jeanne F. Loring, Stephen J. Haggarty

**Affiliations:** 1 Center for Human Genetic Research, Massachusetts General Hospital, Harvard Medical School, Boston, Massachusetts, United States of America; 2 Stanley Center for Psychiatric Research, Broad Institute of Harvard and Massachusetts Institute of Technology, Cambridge, Massachusetts, United States of America; 3 Harvard Stem Cell Institute, Harvard University, Cambridge, Massachusetts, United States of America; 4 Center for Regenerative Medicine, Massachusetts General Hospital, Boston, Massachusetts, United States of America; 5 Center for Regenerative Medicine, Department of Chemical Physiology, The Scripps Research Institute, La Jolla, California, United States of America; National Institutes of Health, United States of America

## Abstract

Fragile X syndrome (FXS) is the most common inherited cause of intellectual disability. In addition to cognitive deficits, FXS patients exhibit hyperactivity, attention deficits, social difficulties, anxiety, and other autistic-like behaviors. FXS is caused by an expanded CGG trinucleotide repeat in the 5′ untranslated region of the Fragile X Mental Retardation (*FMR1*) gene leading to epigenetic silencing and loss of expression of the Fragile X Mental Retardation protein (*FMRP*). Despite the known relationship between *FMR1* CGG repeat expansion and *FMR1* silencing, the epigenetic modifications observed at the *FMR1* locus, and the consequences of the loss of *FMRP* on human neurodevelopment and neuronal function remain poorly understood. To address these limitations, we report on the generation of induced pluripotent stem cell (iPSC) lines from multiple patients with FXS and the characterization of their differentiation into post-mitotic neurons and glia. We show that clones from reprogrammed FXS patient fibroblast lines exhibit variation with respect to the predominant CGG-repeat length in the *FMR1* gene. In two cases, iPSC clones contained predominant CGG-repeat lengths shorter than measured in corresponding input population of fibroblasts. In another instance, reprogramming a mosaic patient having both normal and pre-mutation length CGG repeats resulted in genetically matched iPSC clonal lines differing in *FMR1* promoter CpG methylation and FMRP expression. Using this panel of patient-specific, FXS iPSC models, we demonstrate aberrant neuronal differentiation from FXS iPSCs that is directly correlated with epigenetic modification of the *FMR1* gene and a loss of FMRP expression. Overall, these findings provide evidence for a key role for FMRP early in human neurodevelopment prior to synaptogenesis and have implications for modeling of FXS using iPSC technology. By revealing disease-associated cellular phenotypes in human neurons, these iPSC models will aid in the discovery of novel therapeutics for FXS and other autism-spectrum disorders sharing common pathophysiology.

## Introduction

The autism spectrum disorders (ASDs) are a group of neurodevelopmental diseases caused by multiple genetic and environmental factors [1]. Despite the immense etiological heterogeneity in ASDs, affected individuals have common behavioral manifestations that may arise due to perturbation of common neurodevelopmental processes. In the long term, identification of common cell- and molecular-level elements underlying the ASDs will require a broad study of both idiopathic and genetically correlated cases.

One of the major obstacles to identification of therapeutic interventions for the ASDs has been the difficulty of studying the step-by-step development of the disease in systems that are amenable to drug and functional genomic screening. Recent advances in stem cell biology and the advent of somatic cell reprogramming technology now enable the generation of patient-specific induced pluripotent stem cells (iPSCs) that can be differentiated *in vitro* into a variety of cell types of the nervous system. Through the use of these patient-derived cell models, iPSCs provide a means to: i) potentially recapitulate the step-by-step development of disease, ii) discover the underlying molecular mechanisms involved in the disease pathology, and iii) apply existing and emerging approaches for discovering and testing different classes of therapeutics that target early steps in disease pathogenesis [2]. 

Of the small percentage of individuals with genetically correlated ASD [1], mutations in the X-chromosome linked fragile X mental retardation gene 1 (*FMR1*) gene in Fragile X syndrome are the most prevalent. Clinical manifestations of the syndrome include cognitive abnormalities ranging from mild learning impairment to severe mental retardation which often progressively increases with age [3], [4], [5], [6]. Behavioral symptoms of FXS patients are variable and may include hyperactivity, stereotypic behavior, attention deficits, social difficulties, inappropriate speech, restricted interests, anxiety, and other autistic-like behaviors [4], [5], [6].

Loss of the fragile X mental retardation protein (FMRP) has been shown to be causative for the cognitive and behavioral impairments of FXS [6]. FMRP is a cytoplasmic RNA-binding protein [7] involved in mRNA transport from the nucleus to the dendrites in neurons where it is known to regulate the translation of proteins important for synaptic development and plasticity in an activity-dependent manner [8], [9]. Inactivation of the *FMR1* gene in FXS is caused by expansion of a CGG trinucleotide repeat in its 5′-untranslated region (5′-UTR). Normal individuals have 6-50 CGG-repeats, while carriers of premutations have 50–200 repeats [4]. As a consequence of expansion of the CGG-repeat length in the *FMR1* gene >200, through molecular mechanisms not fully understood [10], the 5-carbon position of cytosine nucleotides linked by a phosphate to guanine nucleotides (CpG dinucleotides) in the promoter and CGG-repeat regions of *FMR1* become hypermethylated, resulting in epigenetic silencing of the gene and loss of FMRP expression. In this manner, FXS can be considered to be an epigenetic disorder and there is growing evidence that the epigenetic state of the *FMR1* gene, rather than the CGG-repeat length itself, is the key determinant of FXS pathogenesis and also treatment response [11], [12], [13], [14].

We report here the development and characterization of iPSC lines from multiple FXS-affected individuals. FXS iPSCs differed from non-diseased control lines in expression and methylation of the *FMR1* gene and phenotypic capacity for *in vitro* neural differentiation. Reprogramming of FXS, but not control fibroblasts, demonstrated an instability of the CGG trinucleotide stretch in the 5′ UTR of the *FMR1* gene. In two cases, we observed that some of the FXS iPSC clones had repeat lengths that were shorter than their corresponding input fibroblasts. In one instance, we produced multiple iPSC clones from a mosaic individual having both normal and pre-mutation length CGG repeats, generating a set of genetically matched iPSC lines differing in their CGG repeat lengths, *FMR1* methylation and in-vitro neural differentiation characteristics. The well-characterized collection of FXS pluripotent stem cells generated in this study will be useful for understanding the mechanisms underlying the disease and for discovery of potential therapeutic interventions.

## Methods

### Human Fibroblast Culture

Fibroblasts from three clinically diagnosed Fragile X Syndrome male patients (GM05848, GM05131 and GM05185) and one unrelated, unaffected male (GM08330) were purchased from Coriell Institute for Medical Research. Fibroblasts from one clinically unaffected male (BJ1-hFib) were also obtained from ATCC. Cells were grown in flasks coated with 0.1% gelatin (EMD Millipore), and grown in fibroblast media: 10% heat-inactivated FBS (Gemini Bio-Products), 1% Penicillin/Streptomycin (Invitrogen), 1% non-essential amino acids (Invitrogen) and 88% DMEM (Invitrogen) filtered through a 0.22 µm bottle-top filter.

### Fibroblast Reprogramming

Retroviruses were generated by tripartite transient transfection of pIK-MLV (gag.pol), pHDM-G (VSV), and the specific pMIG vectors carrying the hOCT4, hSOX2, hKLF4 or hc-MYC genes) into 293T cells as previously described [15], [16]. Fibroblasts were plated in single wells of 6-well plates at 10^5^ cells per well. These cells were then transduced for 24 hours with the four retroviruses with an multiplicity of infection (MOI) of 10 for pMIG-hOCT4-IRES-GFP (Addgene), pMIG-hSOX2-IRES-GFP (Addgene) and pMIG-hKLF4-GFP (Addgene) and MOI of 1 for MCSV-hc-MYC-IRES-GFP (Addgene). After 24 hours, cells were washed with PBS and fresh media was added, and five days later cells were passaged onto 10 cm gelatin-coated dishes with γ-irradiated mouse embryonic fibroblasts (iMEFs) (GlobalStem). The next day the media was changed to iPSC media: 20% Knock-out Serum Replacement ((KOSR), Invitrogen), 1% penicillin/streptomycin (Invitrogen), 1% non-essential amino acids (Invitrogen), 0.5% L-glutamine (Invitrogen), 100 µM 2-mercaptoethanol (Bio-Rad) and 77.5% DMEM/F-12 (Invitrogen) and 10 ng/mL bFGF (Stemgent) filtered through a 0.22 µm filter (EMD Millipore). Dishes had daily media changes until colonies emerged, (3 to 6 weeks after transduction). Colonies were first assessed based on morphology, then for silencing of the retroviral vectors (GFP minus) before being mechanically passaged onto gelatin coated 6-well plates with γ-irradiated mouse embryonic fibroblasts (GlobalStem) as feeders. Using these methods, multiple clones from each line (except GM05185 that produced only one acceptable clone) were chosen for expansion, cryopreservation, and further characterization.

### iPSC Expansion

Reprogrammed colonies were picked into separate wells and grown as separate clones after that point. The first several passages were grown directly on a feeder layer of iMEFs (GlobalStem). For removal of MEFs for downstream RT-PCR and embryoid body formation, iPSCs were grown by indirect co-culture with iMEFs (GlobalStem) on 1:30 Matrigel (BD Biosciences) coated polyethylene terephthalate (PET) inserts with 1.0 µm pore-size in 6-well plates in iPSC media [17], [18].

### Immunocytochemistry

iPSC colonies grown on iMEFs on Permanox Lab-Tek chamber slides (Nunc) were fixed with 10% cold methanol or 4% paraformaldehyde in PBS for 10 minutes. Methanol fixed slides were blocked for 1 hour in PBS plus 5% bovine serum albumin then stained with OCT3/4 (Santa Cruz sc-101534) or NANOG (Abcam ab21624) for 1 hour at room temperature. Paraformaldehyde fixed slides were blocked and then stained with SSEA4 (EMD Millipore MAB4304) or Tra-1–60 (EMD Millipore MAB4360) 1 hour at room temperature. All slides where then rinsed several times with PBS and slides where then incubated at room temperature in appropriate buffer with secondary antibody and Hoechst-33342 for 1 hour at room temperature. After several more rinses, coverslips were affixed with Vectashield (Vector Laboratories) and imaged with a Zeiss Axiovert microscope and 10X objective equipped with a Zeiss Axiocam digital camera.

### In Vitro Differentiation of Embryoid Bodies

To form embryoid bodies**,** iPSC colonies grown by indirect co-culture were broken up and grown in ultra-low attachment 6-well plates (Corning) in iPSC media without bFGF Stemgent and 1% heat-inactivated FBS (Gemini Bio-Products) for a minimum of 19 days. Embryoid bodies were fixed in PBS with 4% paraformaldehyde for 20 minutes and pelleted in low-melt agarose, followed by paraffin embedding and sectioning into 5 µm sections. The sections were mounted on slides and stained with hematoxylin and eosin for morphological examination using an Olympus BX51 microscope with a 40X objective and an Olympus Q Color 5 CCD camera [17].

### Pluripotency Gene Expression Analysis

Cells collected from colonies grown by indirect co-culture were lysed in Trizol (Invitrogen), then mixed with 1/5^th^ volume of chloroform and centrifuged at 200 x g for 5 minutes. The aqueous phase was collected and processed using an RNeasy Mini column (Qiagen) following the Animal Cells protocol. RNA was quantitated by Nanodrop, normalized to 50 ng/µL and reverse transcribed using Qiagen OneStep RT-PCR Kit. Primers for endogenous pluripotency-associated genes were as described [19]: *OCT4/POU5F1* (5′ primer – CTCACCCTGGGGGTTCTATT, 3′ primer – CTCCAGGTTGCCTCTCACTC, 65°C annealing) with a 230 bp product, *REX1* (5′ primer – TCACAGTCCAGCAGGTGTTTG, 3′ primer – TCTTGTCTTTGCCCGTTTCT, 61°C annealing) with a 205 bp product, *NANOG* (5′ primer – CATGAGTGTGGATCCAGCTTG, 3′ primer – CCTGAATAAGCAGATCCATGG, 64°C annealing) with a 192 bp product, and *GAPDH* (5′ primer – AGCCACATCGCTCAGACACC, 3′ primer – GTACTCAGCGGCCAGCATCG, 62°C annealing) with a 302 bp product as a positive control. RT-PCR primers used for transgene analysis were as described [16]: Trans-*OCT4/POU5F1* (5′ primer - CCTCACTTCACTGCACTGTA, 3′ primer - CCTTGAGGTACCAGAGATCT), Trans-*SOX2* (5′ primer - CCCAGCAGACTTCACATGT, 3′ primer - CCTTGAGGTACCAGAGATCT), Trans-*KLF4* (5′ primer - GATGAACTGACCAGGCACTA, 3′ primer - CTTGAGGTACCAGAGATCT), Trans-*c-MYC* (5′ primer - TGCCTCAAATTGGACTTTGG, 3′ primer - CGCTCGAGGTTAACGAATT) all with a 62°C annealing, and using respective vector plasmids as positive control for each.

### Karyotype Analysis

Karyotype analysis was performed by Cell Line Genetics (http://www.clgenetics.com) as previously described [20].

### Neural Differentiation of iPSC Clones

Neural differentiation was initiated from iPSC clones grown under feeder-free conditions to remove iMEFs either by growth directly on Matrigel (BD Biosciences) in mTeSR1 culture medium (StemCell Technologies) or by indirect co-culture with conditioning feeder layers in KOSR medium (Invitrogen) on Matrigel (BD Biosciences) coated 1 µm porosity membrane inserts [17], [18]. Expandable neuronal progenitors were isolated by one of two ways: 1) directly by manual collection of neural rosette structures in mTeSR1 culture media (StemCell Technologies) upon initiation of differentiation by overgrowth of the iPSC colonies; and/or 2) through magnetic-activated cell sorting (MACS) using microbeads conjugated with antibodies to the polysilated form of neural cell adhesion molecule (PSA-NCAM; Miltenyi Biotech). Isolated cells were expanded in neural expansion medium (70% DMEM (Invitrogen), 30% Ham's F-12 (Mediatech) supplemented with B-27 (Invitrogen), 20 ng/ml each EGF (Sigma) and bFGF(R&D Systems) on poly-ornithine (Sigma)/laminin (Sigma) coated culture plates. After five passages in expansion medium, cells were analyzed for NESTIN and SOX1 expression by fixation in 4% paraformaldehyde, followed by primary incubation with rabbit anti-NESTIN polyclonal antibodies (EMD Millipore AB5922) or mouse anti-SOX1 monoclonal antibodies (EMD Millipore AB15766) and subsequent appropriate fluorochrome conjugated secondary antibody for microscopic evaluation. Terminal neural differentiation was achieved by plating expanded cells plated at a seeding density of 40,000 cells per cm^2^ on polyornthine/laminin plates as above in expansion medium lacking both EGF and bFGF, with medium replacement every 3–5 days until the indicated endpoint time. Two wells (6-well plate) were imaged on an IX-Micro automated microscope (Molecular Devices), and 9 images per well were taken (n = 18 images per sample). All in-focus images were selected for analysis. Images were thresholded to a binary image, skeletonized, and then total pixels were counted to determine total process length in each image using ImageJ (http://rsbweb.nih.gov/ij).

### FMR1 Transcript Quantitative RT-PCR

Cells collected from colonies grown by indirect co-culture were lysed in Trizol (Invitrogen) then mixed with 1/5^th^ volume of chloroform and centrifuged at 200 x g for 5 minutes. The aqueous phase was collected and processed using an RNeasy Mini column (Qiagen) and the Animal Cells protocol was followed from Step 4. RNA was quantitated by Nanodrop, normalized to 50 ng/µL, and reverse transcribed by Clontech Reverse Transcription Kit (Clontech) according to manufacturer's instructions. Samples were then run on a Roche LightCycler 480 thermocycler using Roche SYBR Green (Roche Diagnostics) in triplicate in 384-well plates. PCR primers were: *FMR1* (5′primer - CAGGGCTGAAGAGAAGATGG, 3′primer – ACAGGAGGTGGGAATCTGA) with a 174 bp product and *RPL13A* (5′ primer – ACCCTGGAGGAGAGAGGAA, 3′ primer – AGGCAACGCATGAGGAATTA) with a 186 bp product. Transcript levels were then averaged before being normalized using *RPL13A* levels. Fold-change in transcript levels were determined by comparing transcript levels to those from healthy control cells with these values set to 1.

### FMR1 CGG-Repeat Length Analysis

DNA was extracted from live cells using Qiagen DNeasy Blood & Tissue Kit (Qiagen). Isolated DNA was analyzed by Genzyme Genetics (http://www.genzymegenetics.com) using both Southern blot analysis and polymerase chain reaction (PCR) to determine the CGG-repeat length and methylation status of the promoter region of the *FMR1* gene. Southern blot analysis was performed with a ^32^P-labelled probe StB 12.3 on *Eco*RI and *Eag*I digested DNA [21]. PCR products were generated using a fluorescently labeled primer and sized by capillary electrophoresis.

### FMR1 and OCT4/POU5F1 Promoter Methylation Analysis

Bisulfite treatment of genomic DNA purified using Qiagen DNeasy (Qiagen) and pyrosequencing analysis [22], [23] of the *FMR1* and *OCT4/POU5F1* promoters was performed by EpigenDx Inc. (http://www.epigendx.com) using the PSQ™96HS system according to standard procedures with a unique set of primers that were developed by EpigenDx. The human *OCT4/POU5F1* methylation assay covers ten CG dinucleotides in exon 1 region ranging from -50 to +96 from the transcriptional start site based on Ensembl Gene ID Ensembl:ENSG00000204531 and the Transcript ID ENST00000259915. The human *FMR1* methylation assay covers twenty-two CG dinucleotides in the promoter region ranging from -523 to -384 from the transcriptional start site based on Ensembl Gene ID ENSG00000102081 and the Transcript ID ENST00000370475.

### Western Blotting

Cells for immunoblotting were harvested, pelleted, and frozen at −80°C. The pellets were washed three times with PBS with the addition of one Complete Protease Inhibitor Cocktail Tablet (Roche Diagnostics) per 7 mL PBS. Pellets were then resuspended in 200 µL mPER Mammalian Protein Extraction Reagent (Thermo Scientific) with 1∶100 Protease Inhibitor Cocktail III (CalBioChem) and placed on a rocker overnight at 4°C. The samples were centrifuged at 14,000 x g at 4°C for 15 minutes and the supernatants were stored as 50 µL aliquots at −80°C. Protein was quantified using a BCA assay (Thermo Scientific) read on an Envision Plate Reader (PerkinElmer). Equal quantities of total protein were loaded onto 4–15% Criterion pre-cast polyacrylamide gels (BioRad) and run at 100V for 15 minutes, then at 125V for 1.5 hours. Gels were transferred to PVDF membrane (EMD Millipore) using standard procedures at 300 mA for 1 hour. Membranes were rinsed in Tris-buffered saline with Tween (TBS-T), then blocked with TBS-T + 5% powdered nonfat milk for 1 hour. Membranes were transferred to TBS-T with mouse anti-FMRP clone 1C3 (EMD Millipore MAB2160) at room temperature for 2 hours. Membranes were washed 4 times in TBS-T and incubated for 1 hr in horseradish peroxidase-coupled secondary antibody (Cell Signaling Technologies). Membranes were then washed four times in TBS-T, blotted dry, incubated for 5 minutes with SuperSignal West Pico Chemiluminescent Substrate (Thermo Scientific), blotted dry again and exposed to BioMax MR film (Kodak) for band visualization upon development.

## Results

### Characterization of Fibroblasts from Fragile X Donors: Trinucleotide Repeat Length, CpG Methylation, FMR1 mRNA and Protein Levels

**Figure 1:**
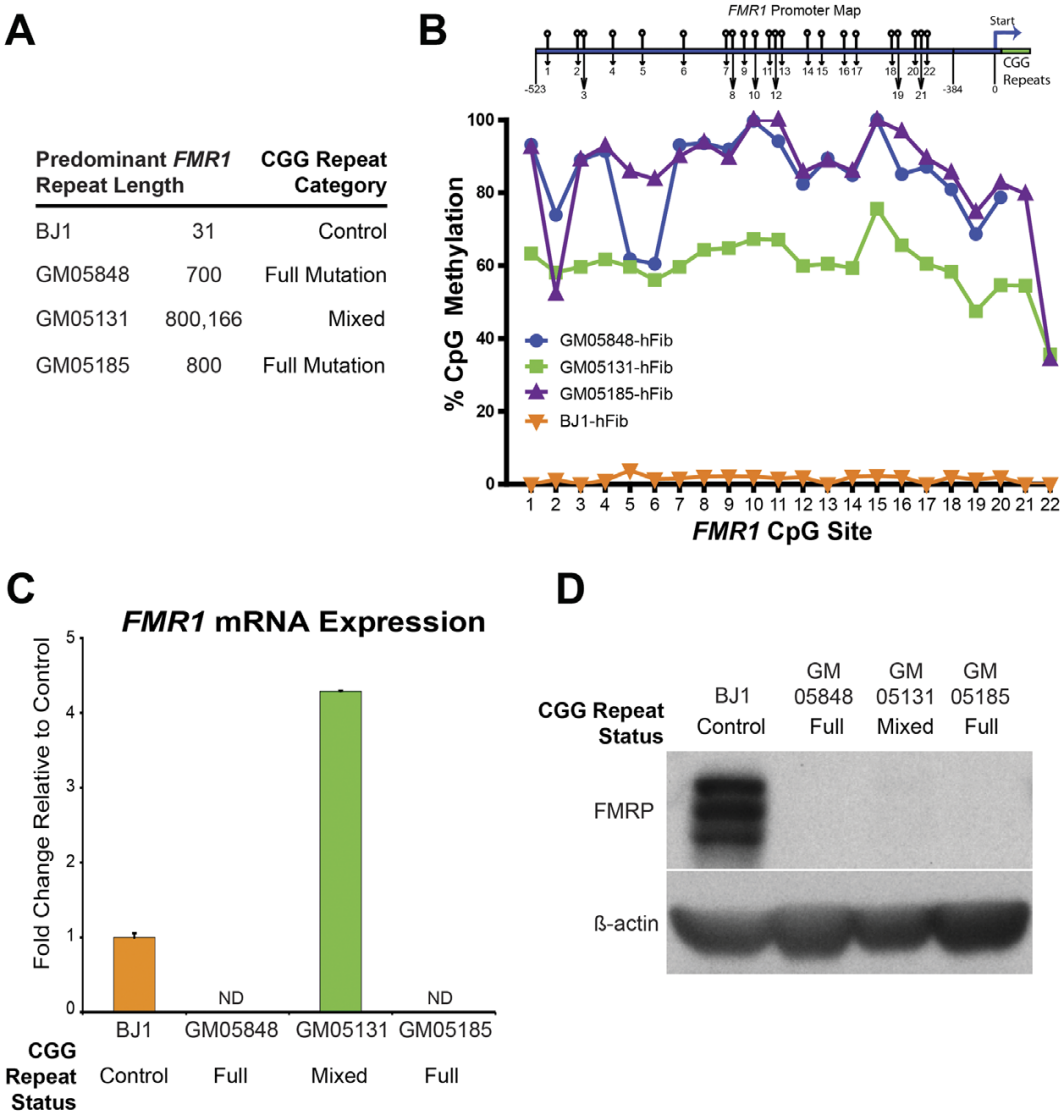
Analysis Fragile X Patient Fibroblasts: *FMR1* CGG-repeat Length, CpG Methylation, and Expression . (A) Predominant CGG-repeat size in the *FMR1* promoter as determined by Southern blot analysis. (B) Bisulphite pyrosequencing analysis of the *FMR1* promoter reported as relative methylation level at indicated CpG positions (*FMR1* promoter CpG site schematic not to scale). (C) *FMR1* transcript expression levels as determined by qRT-PCR shown as fold increase over BJ1 control (ND - non-detectable). (D) Western blot analysis of FMRP protein levels in indicated fibroblast lines, β-actin is shown as a loading control.

Banked fibroblast cell lines (Table 1) were obtained from three clinically typical male FXS patients (Coriell GM05131, GM05185, and GM05848) and two unaffected males (GM08330) and BJ1 (ATCC). As expected, all three FXS patient fibroblast lines (GM05131, GM05185 and GM05848) had CGG repeat sizes in the full mutation range (>200) in the *FMR1* 5′UTR on the X chromosome (Fig. 1A). One of the FXS cell lines, GM05131, however, was shown to be from a mosaic donor, having two predominant bands corresponding to 800 and 166 CGG repeats. Mosaicism in FXS patients has been documented before, and is estimated to occur in 20–40% of patients, possibly due to instability of the repeat length during *in utero* somatic cell development [24]. In the case of fibroblasts derived from GM05131, the fibroblast population was a mixture of cells that have the full mutation and cells with the permutation CGG-repeat lengths.

**Table 1:** Characteristics of FXS patient-derived and healthy control fibroblasts selected for reprogramming.

Cell Line ID	Source	Age at Sampling	Clinical Description
BJ1	ATCC	1 day	Clinically Unaffected
GM08330	Coriell Institute	63 yrs	Clinically Unaffected
GM05848	Coriell Institute	4 yrs	Diagnosed FXS
GM05131	Coriell Institute	3 yrs	Diagnosed FXS
GM05185	Coriell Institute	26 yrs	Diagnosed FXS

Upon increasing CGG-repeat length in the *FMR1* gene beyond the normal range of 6–50, an increase in the methylation of CpG sites in the promoter region leads to epigenetic silencing of the gene [10], [25]. To quantitatively compare the levels of methylation within the promoter region of *FMR1*, bisulfite pyrosequencing was used to query methylation status at 22 CpG sites. Both full mutation fibroblast lines, GM05848 and GM05185 had highly methylated promoter regions, with a mean of approximately 85% of the CpG sites methylated, consistent with the expected hypermethylation of this region in the FXS (Fig. 1B). In contrast, the control BJ-1 fibroblast lines had barely detectable levels (<5%) of CpG methylation in the same region. The mean CpG methylation level of this region in the mosaic GM05131 fibroblast cell line was approximately 60%, most likely because of the presence of both cells with a hypermthylated CpG full mutation as well as premutation fibroblasts with unmethylated *FMR1* promotor regions.

The combination of an expanded 5′-UTR CGG trinucleotide repeat along with the high degree of CpG site methylation of this region would be expected to result in the silencing of the expression of the *FMR1* gene. In order to test this directly, we performed quantitative RT-PCR analysis to assess the relative *FMR1* expression levels in the cell lines (Fig. 1C). While the control lines expressed the *FMR1* gene, the full mutation fibroblast lines had undetectable levels of *FMR1* mRNA expression. Interestingly, the expression level of the *FMR1* gene in the mosaic (premutation plus full mutation) GM05131 line was approximately four-fold higher than the control. This is consistent with reports that the presence of the premutation causes an increase in gene expression from the *FMR1* promoter from two- to ten-fold over unaffected controls [26].

FMRP protein expression in the patient fibroblasts was determined by Western blot analysis (Fig. 1D). Whereas FMRP was highly expressed in the unaffected control line, FMRP protein expression was not detectable in any of the FXS patient fibroblasts. Interestingly, the mosaic GM05131 fibroblasts that demonstrated only partial methylation of the *FMR1* promoter had undetectable protein, even though they had elevated transcript levels; it has been reported before that production of FMRP is greatly reduced in the premutation state, which may be due in part to a relative block in translation caused by the presence of the 5′UTR extended CGG repeat [27].

### Derivation and Characterization of FXS Induced Pluripotent Stem Cells

**Figure 2:**
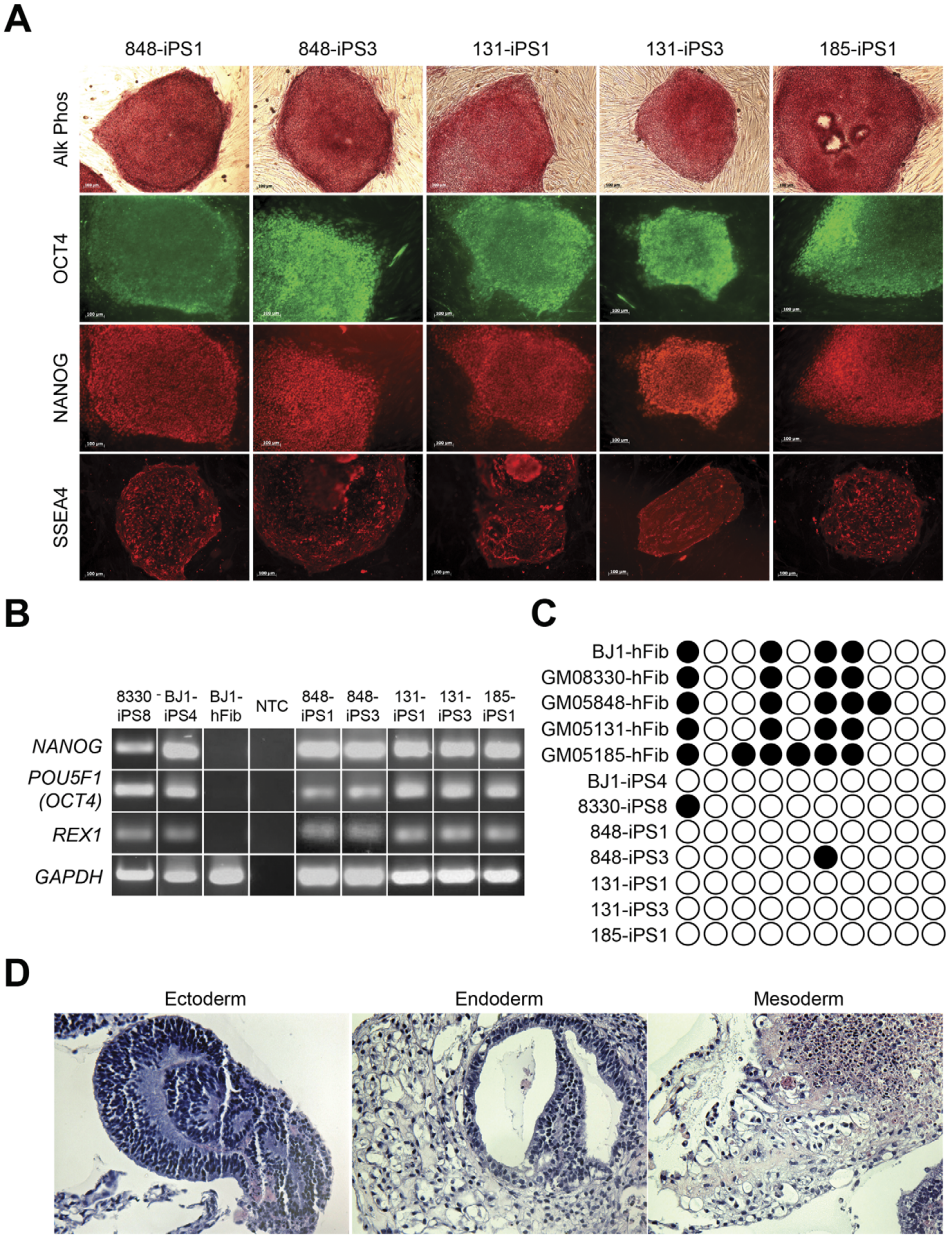
Derivation and Characterization of FXS Induced Pluripotent Stem Cells. (A) Alkaline phosphatase enzymatic and pluripotent marker (OCT4, NANOG and SSEA-4) immunocytochemical analysis of FXS patient-derived iPSC clones. (B) Endogenous *OCT4*, *NANOG* and *REX1* pluripotency-associated transcript expression as analyzed by RT-PCR in indicated iPSC lines and fibroblasts (NTC - non-template containing control). (C) Bisulphite pyrosequencing analysis of the endogenous *OCT4/POU5F1* promoter in indicated lines (open circles, unmethylated (<50%) CpGs; black circles, methylated CpGs). (D) Embryoid body pathological evaluation of H&E stained sections (clone 848-iPS1 shown) indicating representative ectoderm (neural epithelium, left), endoderm (respiratory epithelium, center) and mesoderm (connective tissue, right) germ layers.

FXS patient and control fibroblasts were reprogrammed to pluripotency using established methods (see Methods) [16], [28]. We further analyzed two iPSC clones from GM05848 (referred to as clones 848-iPS1 and 848-iPS3), two clones from GM05131 (131-iPS1 and 131-iPS3), one clone from GM05185 (185-iPS1) and control iPSC lines from GM08330 (8330-iPS8) and BJ1 (BJ1-iPS4). All iPSC clones had typical characteristics of human pluripotent stem cells indicating successful reprogramming (Fig. 2), including: a) human embryonic stem cell colony-like morphology, b) alkaline phosphatase expression and immunoreactivity for OCT4 (POU5F1), NANOG and Stage-specific embryonic antigen-4 (SSEA-4) (Fig. 2A), c) expression of endogenous *OCT4*, *NANOG*, and *REX1* (Fig. 2B), d) de-methylation of the endogenous *OCT4* promoter (Fig. 2C), and e) normal karyotypes (data not shown) [29]. In addition, both FXS and control iPSC clones differentiated into all three germ layers *in vitro* (Fig. 2D) [30], including early neural tissue. Importantly, in concordance with the assessment of a loss of GFP expression from the retroviral vectors, analysis of transgene expression in the control and Fragile X syndrome iPSC clones using RT-PCR and primers specific for transgene *cMYC*, *OCT4/POU5F1*, *KLF4*, *SOX2* indicated a silencing of their expression (Fig. S2). Observation of the growth rate and the ability to remain undifferentiated in culture over many passages (>20) did not reveal any obvious qualitative differences between the unaffected control and FXS iPSC lines.

### Generation of Both FXS Full Mutation and Unaffected iPSCs from a Mosaic Culture

**Figure 3:**
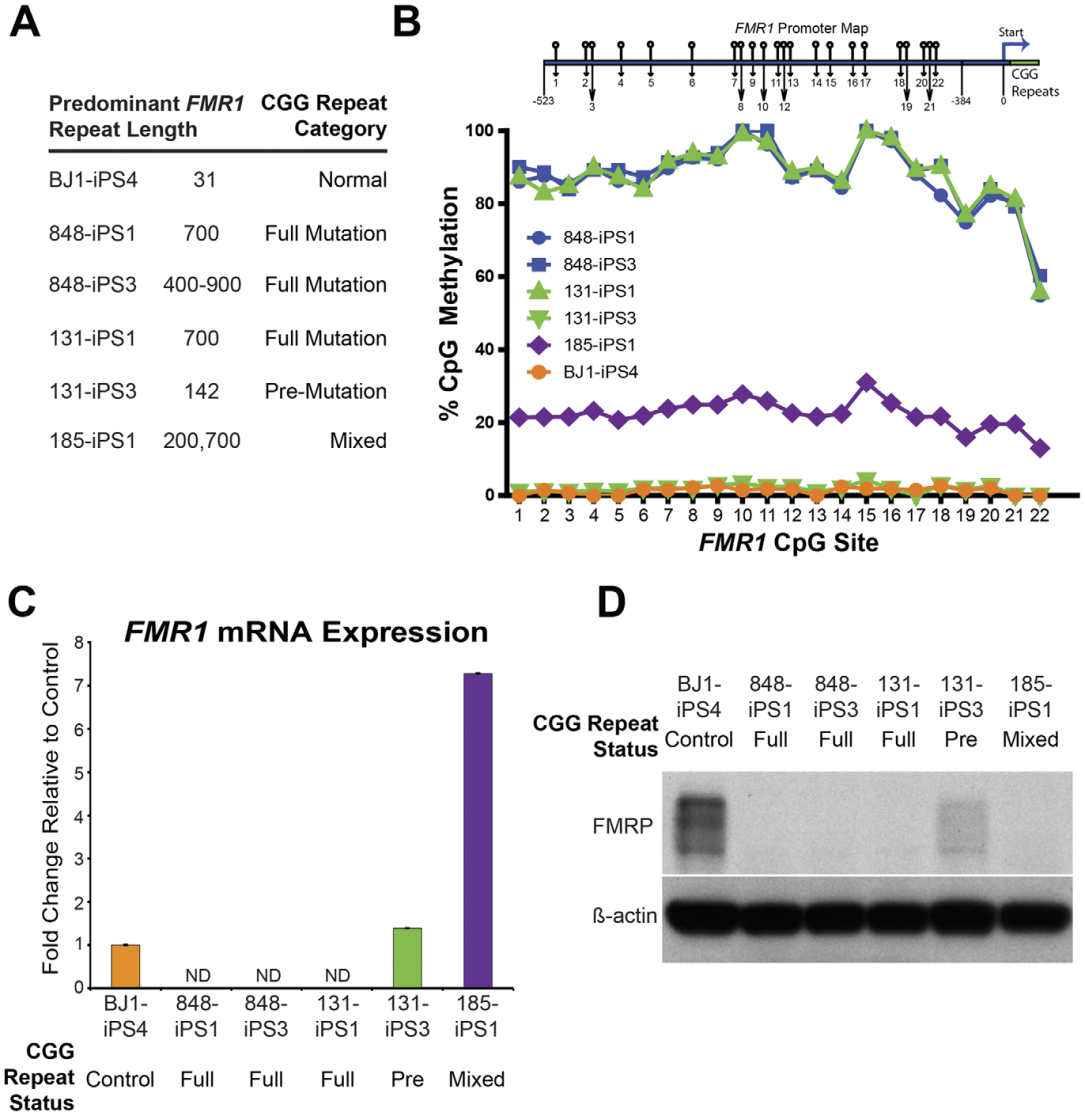
Effects of iPSC Generation on *FMR1* CGG-repeat Length, CpG Methylation and Expression. (A) Predominant CGG-repeat length in the *FMR1* promoter as determined by Southern blot analysis. (B) Pyrosequencing analysis of the *FMR1* promoter reported as relative methylation level at indicated CpG positions (*FMR1* promoter CpG site schematic not to scale). (C) *FMR1* transcript expression levels as determined by qRT-PCR shown as fold increase over BJ1 control (ND - non-detectable). (D) Western blot analysis of FMRP protein levels in indicated iPSC lines, β-actin was used as a loading control.

We found that the two iPSC clones we generated from the GM05131 cell line appear to be derived from the two different fibroblast subpopulations. One iPSC clone had approximately 700 and the other 140 CGG repeats (Fig. 3). These CGG-repeat lengths are similar to those detected in the heterogeneous input fibroblasts (Fig. 3A). Characterization of methylation of the *FMR1* promoter region showed that, as expected, the iPSC clone 131-iPS1 (CGG-repeat length of 700) had a mean CpG methylation of approximately 90%, while clone 131-iPS3 (142 CGG repeats) was essentially unmethylated (Fig. 3B). *FMR1* expression analysis showed that there were no detectable transcripts from the fully CpG methylated 131-iPS1 clone, while the premutation 131-iPS3 clone showed increased expression compared to the unaffected controls (Fig. 3C). The distinctive difference between these two clones shows that derivation of iPSC resulted in clonal selection of these two subpopulations from the mosaic population.

FMRP protein expression differed in the full- and pre-mutation iPSC clones (Fig. 3D). The full mutation 131-iPS1 line did not show detectable FMRP expression, whereas the 131-iPS3 premutation line showed low, but detectable, levels of FMRP. Thus, the premutation clone 131-iPS3 had a combination of increased *FMR1* mRNA expression and decreased protein production; this phenomenon has been observed before [26], [27], and the reduced protein-to-mRNA ratio has been attributed to reduced translational efficiency of the premutation transcript.

### Reprogramming Effects on Trinucleotide Repeat Length, FMR1 Expression and CpG Methylation

While the trinucleotide repeat lengths in the mosaic fibroblast population appeared to remain similar after reprogramming, in several cases iPSC lines derived from FXS fibroblast lines had *FMR1* CGG-repeat lengths that were clearly different from the original fibroblasts (Fig. 3A). The two FXS GM05848-derived iPSC clones had different predominant CGG-repeat lengths: clone 848-iPS1 had the expected 700 CGG repeats, coinciding closely to that of the input fibroblasts, but the other clone from this fibroblast line, 848-iPS3, showed a range of CGG-repeat lengths ranging from 400–900 repeats (Fig. 3A). This suggests that at some point during the reprogramming process, or subsequent expansion, the CGG repeats became unstable in this clone. We detected no changes in the CpG methylation status of the promoter compared to the input fibroblasts (Fig. 3B) and there was as no detectable *FMR1* transcript expression (Fig. 3C).

We observed a different type of repeat length change in the FXS GM05185-derived iPSCs. The fibroblast line contained approximately 800 CCG repeats as determined by Southern blot analysis, but after reprogramming the 185-iPS1 line had two different discrete predominant lengths of approximately 200 and 700 CGG repeats. Since these are male cells, the existence of two bands from an X-linked gene indicates that this iPSC line contained two distinctly different subtypes. Promoter CpG methylation analysis showed a mean of 22% in these cells (Fig. 3B), in contrast to the GM05185 fibroblasts, which were almost completely methylated in the CpG site in this region. This methylation content is likely to be due to contributions from the two populations, one highly methylated and the other relatively unmethylated. The 200 CGG-repeat length is indicative of the premutation state, so it would be expected that *FMR1* transcription would be increased relative to fully mutated and control cells. Indeed, the mRNA level in 185-iPS1 cells was several fold higher than controls (Fig. 3C). These cells also showed a lack of FMRP protein production, indicating defective translation of the premutation transcript.

The source of the 200-repeat iPSC subpopulation is not clear. There was no premutation population detected in the fibroblast cultures and the fibroblasts had no detectable *FMR1* transcript expression. It is possible that the reprogramming selected for a rare undetectable premutation subpopulation within the fibroblast culture. Because we also observed changes in repeat length in the 848-iPS3 FXS iPSC line, it is also possible that the reprogramming process itself led to CGG-repeat length shortening.

### Aberrant Neural Differentiation from FX iPSCs is Dependent on CGG Repeat Length and FMR1 Methylation

Since ASDs are neurodevelopmental disorders, we investigated the effects of FXS mutations on iPSC differentiation along a neural lineage. Previous reports have been inconsistent about the effects of FXS on neuronal differentiation; one study [31], reported that *in vitro* differentiation of neurospheres derived from post-mortem human FXS brain and unaffected fetal brain showed differences in morphology, neurite number and length, and an altered ratio of Tuj1-positive to glial fibrillary acidic protein (GFAP)-positive cells; another similar study saw no significant differences in neural differentiation between FXS and control cells [32].

**Figure 4:**
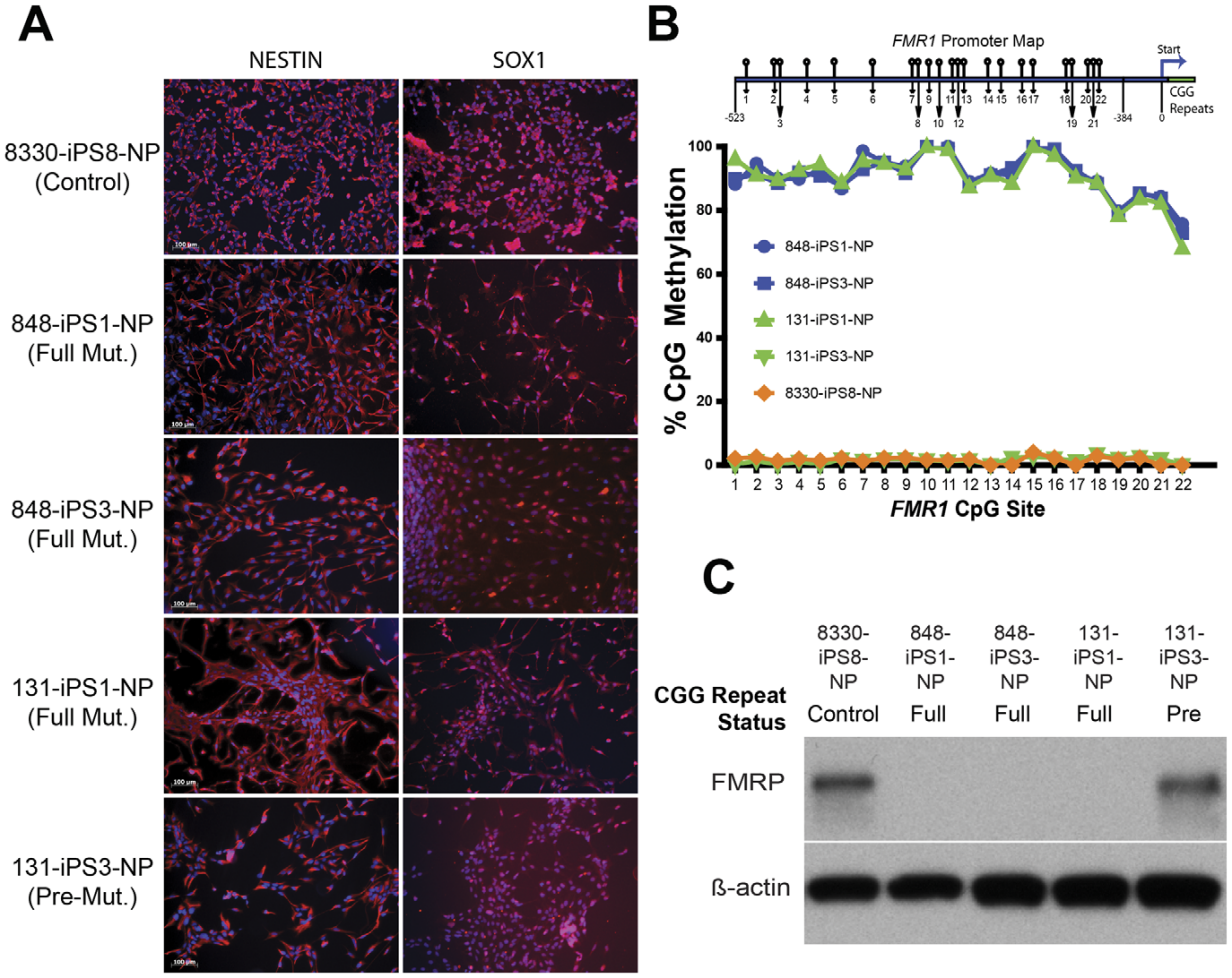
Isolation and Characterization of Expandable Neuronal Progenitor Cells from iPSC Clones. (A) Immunocytochemical analysis of NESTIN and SOX1 (red, nuclei DNA staining overlaid in blue) expression in expanded neural cells from indicated iPSC lines expanded in the presence of mitogens EGF and bFGF. (B) Bisulphite pyrosequencing analysis of the *FMR1* promoter reported as relative methylation level at indicated CpG positions in indicated neural differentiated iPSC lines (*FMR1* promoter CpG site schematic not to scale). (C) Western blot analysis of FMRP protein levels in indicated neural differentiated iPSC lines, β-actin is shown as a loading control.

**Figure 5:**
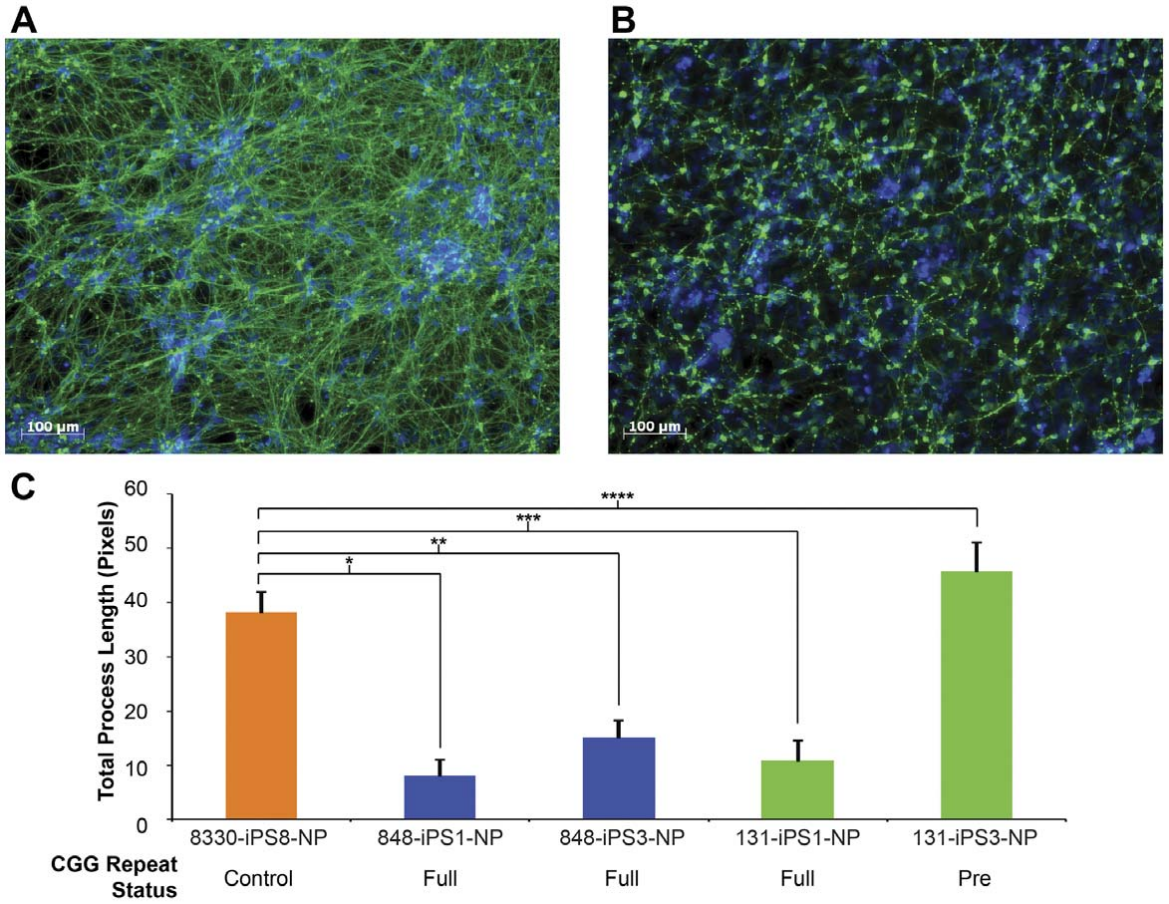
Aberrant Neural Differentiation of FXS iPSC-derived Neuronal Progenitors. Immunocytochemical analysis of Tuj1 expression (green) in neuronal progenitor cells differentiated upon mitogen removal of (A) FMRP^+^ control line 8330-8 and (B) FMRP^-^ FXS line 848-3, overlaid with nuclei DNA staining (blue) (see Figure S1 for additional images). (C) Quantification of neurite process length in indicated iPSC-NP lines. Two wells (6-well plate) were imaged, 9 images per well (n = 18 images per sample). P-values as indicated *1.5e-13, **1.1e-10, ***3.1e-13, ****1.4e-4.

**Figure 6:**
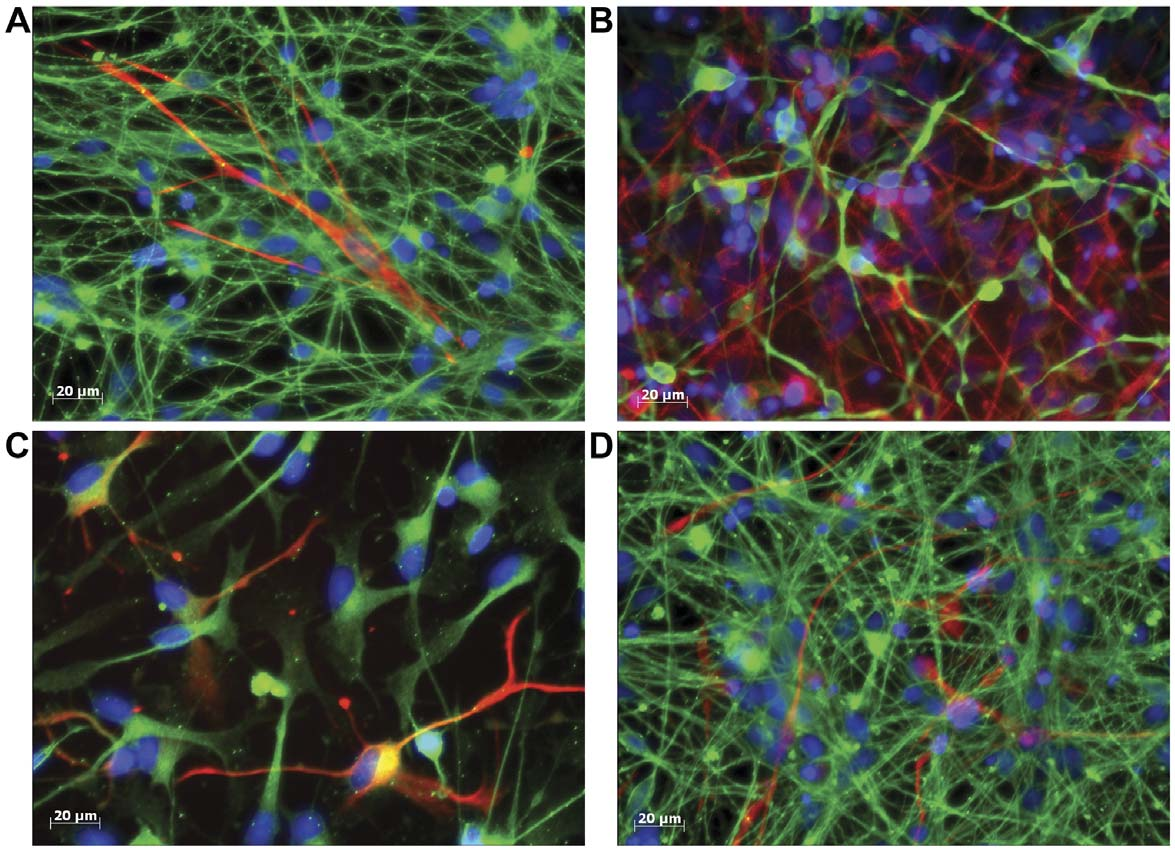
Rescue of Aberrant Neural Differentiation of FXS iPSC-derived Neuronal Progenitors by FMRP Expression in Premutation Clones. Immunocytochemical analysis of expanded neuronal progenitor cells differentiated upon mitogen removal of FMRP^+^ control line 8330-8 (A) and FMRP^-^ FXS lines 848-3 and 131-1 (B and C) and FMRP^+^ FXS line 131-3 (D), overlay of Tuj1 staining (green), GFAP (red) and nuclei DNA staining (blue) (see Figure S1 for additional images).

We compared neural differentiation from clones with high *FMR1* CpG methylation (848-iPS1, 848-iPS3, and 131-iPS1) with that of unmethylated clones (131-iPS3 and 8330-iPS8). We generated NESTIN+ and SOX1+ expandable neural progenitor cells and characterized their *FMR1* promoter CpG methylation status and FMRP expression levels, which were observed to closely correspond to the iPSC clones from which they were derived (Fig. 4). We then analyzed differentiated iPSC-derived progenitor cells after withdrawal of mitogenic factors (EGF, bFGF) using immunocytochemistry for lineage-specific markers Tuj1 (neural) and GFAP (glial). We found that all of the tested iPSC-derived progenitor lines could be induced to form neurons and glia (Fig. 5 and Fig. S1). However, there was a notable difference between the FXS and control cells in the number and length of the processes of Tuj1-positive cells (Fig. 5 and Fig. S1). The control cells (*FMR1* unmethylated, 8330-iPS8 and 131-iPS3) had extensive long and highly branched processes, while the FXS iPSC-derived cells (*FMR1* methylated; 848-iPS1, 848-iPS3, and 131-iPS1) exhibited fewer and much shorter processes (Fig. 5 and Fig. S1). The FXS cells also appeared to be flatter and have only a single process. It is of interest to note that the two subclones (131-iPS1 and 131-iPS3) derived from the mosaic donor differed in their neural differentiation in spite of their presumed common genetic background (Fig. 6).

Glial cells also differed in the differentiated cultures (Fig. 6 and Fig. S1 F–J). The unmethylated control line 8330-iPS8 generated only a few GFAP-positive cells with long processes, which were distributed in patches throughout the cultures. One of the FXS lines, 131-iPS1 was similar to the control (Fig. 6 and Fig. S1 F–J). In contrast, the cultures derived from the two 848-iPSC FXS lines (848-iPS1 and 848-iPS3) consistently had a larger number of GFAP-positive cells with more compact morphology (Fig. 6B and Fig. S1 G,H,L and M). However, one of the FXS lines, 131-iPS1, more closely resembled control lines in its GFAP expression (Fig. 6C and Fig. S1 I and N). These observations suggest that glial phenotypes may be more variable among different FXS patient-derived iPSC models and that factors other than *FMR1* affect glial, but not neuronal differentiation.

## Discussion

Fragile X Syndrome (FXS) is one of a group of genetic diseases that are caused by pathogenic expansion of a trinucleotide repeat. The diseases, including Huntington's disease and Friedreich's ataxia, are characterized by neurological dysfunction, often in specific regions of the CNS. The trinucleotide repeats disrupt specific genes in each disease, but very little is known about how the expansion reaches pathological levels and how the dysfunction of specific genes leads to neurological disorder. In some cases, such as Huntington's disease, the expanded repeat is exonic and causes the expression of a pathogenic protein [33]. In FXS and Friedreich's ataxia [14], the repeat is in a non-coding region of the gene and the pathogenic expansion results in silencing of the gene through epigenetic mechanisms. In FXS, the disease is triggered when there are 200 or more copies of a CGG trinucleotide repeat in the 5′UTR of the *FMR1* gene. The number of repeats predicts the pathology: unaffected individuals have about 30 repeats, the *FMR1* gene promoter is unmethylated, and the FMRP protein is expressed; full mutation individuals (>200 repeats) have fully methylated *FMR1* and produce no protein [10]. Interestingly, the intermediate premutation (ca. 100–150 copies) has elevated transcription but translation of the FMRP protein is inefficient [26], [27]. Premutation carriers, while having normal intelligence, demonstrate a range of psychiatric and behavioral symptoms and are associated with a number of medical conditions such as Fragile X-associated tremor/ataxia (FXTAS) and Premature Ovarian Insuffuciency (POI) in response to the elevated transcription levels of *FMR1* [34].

Trinucleotide repeat diseases have been difficult to study because of limitations in availability of cells and tissues from the brains of affected individuals. Mouse models of these diseases are suboptimal because of differences in neural development in mouse and human. The development of iPSC technology has enabled *in vitro* studies of central nervous system cells derived from patients with genetic neurological disease. However, the value of iPSC modeling of human disease relies on the assumption that the resulting iPSC lines contain the same causative elements of the disease that the input patient cells contained. The data we present here draw into question this assumption, and show that the iPSCs derived from FXS individuals do not necessarily faithfully reproduce the CGG-repeat lengths, CpG methylation status, and silencing of the *FMR1* gene in the fibroblasts of origin. We also show that differences in neuronal differentiation among FXS iPSC lines are attributable at least in part by the epigenetic status of the *FMR1* gene promoter.

Existing mouse models with a knock-out of *Fmr1* are not appropriate for investigating questions of repeat stability or the epigenetic mechanisms of *FMR1* silencing as they lack the expanded trinucleotide repeat. Knock-in mouse models in which the murine CGG repeat has been replaced with a premutation-sized CGG repeat from humans were reported to exhibit moderate repeat instability with both paternal and material transmission [35], [36]. However, in addition to CGG-repeat length, since the nature of the flanking sequences in combination with the patterns of interruption of CGG repeats can influence nucleosomal structure and alter CGG repeat instability [37], [38], the use of genetically accurate, human neuronal models will be advantageous to investigate the molecular mechanisms of trinucleotide repeat instability and epigenetic regulation.

In one case, we discovered that a patient fibroblast cell line, GM05131, is a heterogeneous mixture of normal and full FXS mutation cells. Reprogramming of these fibroblasts resulted in two iPSC clones, one with the full FXS mutation (ca. 700 CGG repeats) and the other with premutation repeat length (ca. 142 repeats). As expected, the full mutation cells produced no *FMR1* transcript and the premutation clone had above-normal *FMR1* transcription levels but very low translation of the FMRP protein. These two clones are presumably otherwise genetically matched, which will be valuable for comparison of the effects of the full- and premutation in the absence of potentially confounding background genetics. Interestingly, the CGG-repeat lengths in the iPSCs appeared to be slightly shorter than those of the fibroblasts (700 vs. 800; 142 vs. 166); in light of the similar changes that appeared upon reprogramming of the other fibroblast lines (see below), it seems possible that the reprogramming process may lead to instability of trinucleotide repeat lengths.

A second FXS line, GM05848 (ca. 700 CGG repeats), gave rise to an iPSC clone that apparently possessed multiple trinucleotide repeat lengths ranging from 400 to 900. And the third FXS line reprogrammed (GM05185) had a predominant trinucleotide repeat of 800, but produced a heterogeneous iPSC line (185-iPS1) with discrete repeat lengths (200 and 700). There was no apparent heterogeneity in the input fibroblasts, as evidenced by lack of *FMR1* transcript detected, even after extensive qRT-PCR (40–45 rounds). The full mutation iPSC clones (848-iPS1 and -iPS3) showed no detectable *FMR1* expression, but the 185-iPS1 clone, with a *de novo* premutation subpopulation present, expressed the high levels of FMR1 transcript typical of premutation cells.

These data suggest that reprogramming of full mutation FXS fibroblasts results in changes, generally shortening, of the repeat length in the resulting iPSC clones. A varied population of repeat lengths in one isolated iPSC clone (848-iPS3) suggests that change in CGG-repeat length is a dynamic process that occurs because of instability of the CGG repeat initiated during reprogramming. An earlier study reported that full mutation human *FMR1* alleles stably maintained in patient fibroblasts and murine A9 somatic hybrid cells contracted upon transfer to pluripotent embryocarcinoma (PC13) cells due to instability upon passage [39]. Repeat instability is also suggested by the variable repeat lengths observed in a human embryonic stem cell line derived from a FXS-affected embryo, which showed repeat length heterogeneity from 200 to more than 1,000 triplet CGG repeats in the same isolated clone [19].

This is the first report of trinucleotide repeat length change in FXS iPSCs. A previous analysis of FXS iPSCs did not report trinucleotide repeat length changes [40]. However, changes in repeat length with reprogramming has been reported for another trinucleotide repeat disease, Friedrich's ataxia [41]; in that case, in iPSCs there was an expansion of an intronic GAA repeat that silences the *FXN* gene on chromosome 9. That report and the current study suggest that reprogramming may destabilize repeats in certain trinucleotide repeat diseases. Further investigation of this phenomenon may help in understanding the basis of transgenerational instability of pathological trinucleotide repeat sequences in many neurodevelopmental diseases.

The impact of repeat instability on iPSC *in vitro* models of FXS could be considerable if the iPSC repeat length is not determined. We found that in general the actual repeat length in the iPSCs predicted the methylation status and expression levels of FMRP transcripts and proteins, and therefore the disease state, regardless of the status of the input fibroblasts. If the changes in repeat length are truly dynamic, researchers may find unexpected phenotypes in iPSC derivatives if they do not monitor the repeat length in the cells.

Two previous reports have investigated *FMR1* expression in human pluripotent cells, with conflicting results: one study used FXS human embryonic stem cells (hESCs) [19] and the second studied FXS iPSCs [40]. The first report indicated that the *FMR1* gene was expressed in the FXS-hESCs, despite the cells having full mutation status, and was repressed only after differentiation [19]. The second study reported that *FMR1* expression was repressed in both full mutation undifferentiated FXS-hESCs and FXS patient-derived iPSCs (from the GM05848 line) [40]. Our results support the report on FXS iPSCs; we observed promoter CpG methylation and *FMR1* repression in GM05848-derived iPSCs as well as in all other iPSC clones that contained only full mutation alleles. We also characterized neuronal differentiation in several FXS iPSC lines, showing for the first time that the CpG methylation state of the *FMR1* gene in iPSCs persists during neuronal differentiation, an observation that is critical for efforts to use iPSC-derived cells to model FXS.

We observed FXS-associated morphological differences in iPSC-derived neurons, with FXS cells having fewer and shorter neurites than controls. Similar neuronal morphology has been reported in *FMR1* knock-out mouse models [42], [43] and post-mortem fetal FXS brain tissue [31], [44]. The morphological differences correlated with *FMR1* promoter CpG methylation status and expression of *FMR1,* and occurred in multiple iPSC lines from different source fibroblasts. We also observed variations in glial differentiation as assessed by GFAP immunostaining, although these phenotypes were not strictly linked to *FMR1* methylation status. There have been previous reports of differences in glial/neuronal ratios in FXS-derived cell cultures. Adult neural stem cells from the dentate gyrus of *Fmr1* knockout mice showed increased glial differentiation as compared to controls [43]. Observations using human neural tissue differ and are possibly brain region-specific; neurospheres derived from FXS hippocampal tissue showed reduced glial differentiation [31], [44], whereas cortex-derived cells were unaffected [32].

Overall, our results suggest an important role for FMRP early in human neurodevelopment. In this context, future studies will be aimed toward understanding the molecular basis of the observed phenotypes and exploring the consequence of a loss of FMRP on signaling and synaptic function in FXS–derived neuronal cells. Having identified a robust, morphological phenotype upon neural differentiation of FXS iPSCs provides an opportunity for the characterization of existing pharmacological agents and to potentially discover novel therapeutics that can reverse disease-associated phenotypes in FXS and other ASDs sharing common pathophysiology.

## Supporting Information

Figure S1 **Analysis of Tuj1 and GFAP Expression Upon Post-mitotic Neural Differentiation.** Immunocytochemical analysis of expanded neural cells differentiated upon mitogen removal. (A to E) Tuj1 staining (100X) indicated in green. F to J) GFAP staining (100x) indicated in red. (K to O) Tuj1(green) and GFAP (red) overlay (400X). In all images, nuclei DNA co-staining is indicated in blue.
(TIF)

Figure S2 **RT-PCR Verification of Reprogramming Transgene Silencing of GFP-minus iPSC Clones.** Transgene specific RT-PCR demonstrates silencing of retroviral–specific reprogramming genes (*OCT4/POU5F1*, *SOX2*, *KLF4* and *cMYC*) in indicated iPSC lines using respective vector plasmids as positive control for each.
(TIF)
