# Supplementary figures and images for: Epigenetic Characterization of the FMR1 Gene and Aberrant Neurodevelopment in Human Induced Pluripotent Stem Cell Models of Fragile X Syndrome
Source: PLoS One. 2011 Oct 12;6(10):e26203. doi: 10.1371/journal.pone.0026203 (PMC3192166; doi:10.1371/journal.pone.0026203)

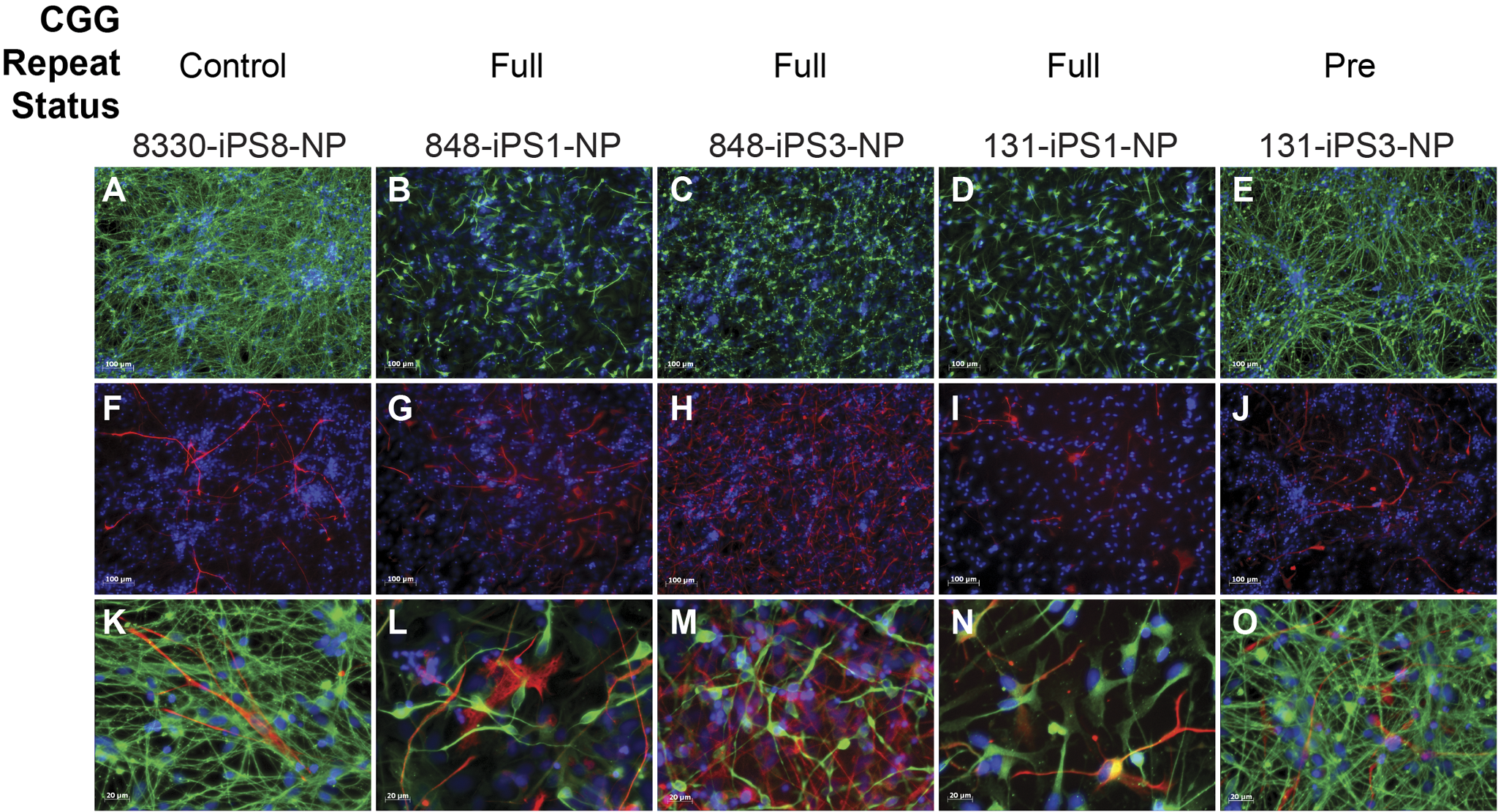

Supplement: Figure S1 — Analysis of Tuj1 and GFAP Expression Upon Post-mitotic Neural Differentiation. Immunocytochemical analysis of expanded neural cells differentiated upon mitogen removal. (A to E) Tuj1 staining (100X) indicated in green. F to J) GFAP staining (100x) indicated in red. (K to O) Tuj1(green) and GFAP (red) overlay (400X). In all images, nuclei DNA co-staining is indicated in blue. (TIF) [file pone.0026203.s001.tif]

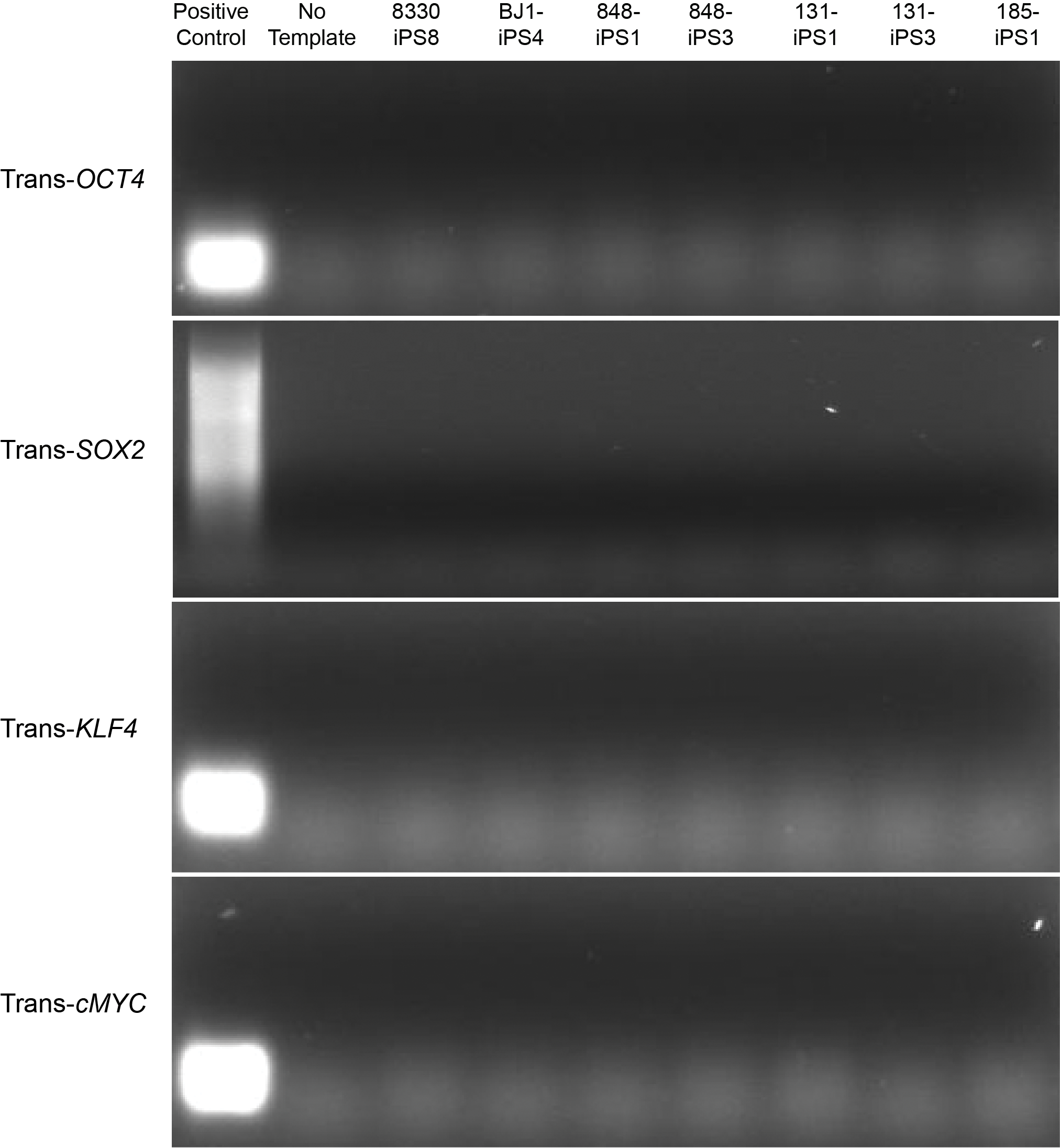

Supplement: Figure S2 — RT-PCR Verification of Reprogramming Transgene Silencing of GFP-minus iPSC Clones. Transgene specific RT-PCR demonstrates silencing of retroviral–specific reprogramming genes (OCT4/POU5F1, SOX2, KLF4 and cMYC) in indicated iPSC lines using respective vector plasmids as positive control for each. (TIF) [file pone.0026203.s002.tif]
